# *Bifidobacterium animalis* MSMC83 Improves Oxidative Stress and Gut Microbiota in D-Galactose-Induced Rats

**DOI:** 10.3390/antiox11112146

**Published:** 2022-10-29

**Authors:** Porntipha Vitheejongjaroen, Anongnard Kasorn, Narathip Puttarat, Fabien Loison, Malai Taweechotipatr

**Affiliations:** 1 Center of Excellence in Probiotics, Srinakharinwirot University, Bangkok 10110, Thailand; 2 Department of Microbiology, Faculty of Medicine, Srinakharinwirot University, Bangkok 10110, Thailand; 3 Department of Biomedical Science, Faculty of Medicine, Vajira Hospital, Navamindradhiraj University, Bangkok 10300, Thailand; 4 Department of Microbiology, Faculty of Science, Mahidol University, Bangkok 10400, Thailand; 5 Systems Biology of Diseases Research Unit, Faculty of Science, Mahidol University, Bangkok 10400, Thailand

**Keywords:** probiotics, *Bifidobacterium*, oxidative stress, inflammation, microbiota

## Abstract

The development of many chronic diseases is associated with an excess of free radicals leading to harmful oxidative stress. Certain probiotic strains have been shown to have antioxidant and anti-aging properties and are an important resource for development of microbial antioxidants. The present study aimed to explore the protection offered by *Bifidobacterium animalis* strain MSMC83 in a model of oxidative stress induced by D-galactose (D-gal). Male Sprague Dawley rats were randomly allocated to four groups: a control group injected with saline, a group injected subcutaneously with D-galactose, a probiotic group injected with D-galactose and administered *B. animalis* MSMC83 (10^9^ CFU/mL) via daily oral gavage, and an ascorbic acid group. The probiotics significantly increased the superoxide dismutase, catalase, and glutathione peroxidase and significantly decreased the malondialdehyde in the plasma and livers of D-galactose-treated rats. Moreover, tumor necrosis factor-alpha level in the liver was significantly decreased. Furthermore, the treatment with *B. animalis* MSMC83 restored the microbiota diversity after D-galactose injection. Therefore, our results supported a beneficial role of *B. animalis* MSMC83 in alleviating oxidative stress through the increased expression of antioxidant enzymes and reduction of pro-inflammatory cytokines in rats. Our study suggests that *B. animalis* MSMC83 may be part of a healthy diet to prevent oxidative stress-associated diseases.

## 1. Introduction

Oxidative stress is an imbalance between excessive reactive oxygen species (ROS) levels and low levels of antioxidant activity [1]. ROS, as free radicals, are byproducts of endogenous oxidation–reduction reactions in normal metabolic processes. However, cell stimulation by external factors, such as cigarette smoke or ultraviolet radiation, results in ROS levels exceeding the cellular antioxidant mechanisms [2]. Excess ROS interact with various biomolecules, resulting in dysfunction and cellular damage, leading in turn to structural and functional disorders [3]. Severe oxidative stress has been associated with human diseases such as diabetes mellitus, heart failure, rheumatoid arthritis, inflammatory bowel diseases, colon cancer, Alzheimer’s disease and aging [4]. Tumor necrosis factor-alpha (TNF-α) has a key role as a cytokine that initiates and promotes inflammation [5]. The inflammatory process is a common source of oxidative imbalance causing oxidative stress and reducing the antioxidant capacity of cells. As the central organ responsible for detoxification and nutrient metabolism, the liver is particularly exposed to oxidative stress. The deleterious effects of free radicals are usually counterbalanced by the production of antioxidants [6]. These antioxidants, including enzymes and small peptides, reduce the redox reactions in the cells and inhibit free radicals from the redox chain reaction [7]. In humans, the primary antioxidants are superoxide dismutase (SOD), glutathione peroxidase (GSH-Px), and catalase. When the body does not produce enough antioxidants for the protection of biological macromolecules, it is necessary to increase antioxidant levels to protect against oxidative damage [8]. Some studies have shown that probiotics including *Lactobacillus* and *Bifidobacterium* have antioxidant properties and reduce oxidative stress [9]. Moreover, the *Bifidobacterium* genus was shown to be anti-aging, anti-inflammatory, and able to modulate oxidative stress [10]. Probiotics, as part of the gut microbiome, are important in microbial balance, reducing inflammation and oxidative stress, resulting in the maintenance of gut health [11]. However, whether these properties are shared by all the genus’ species present in the microbiota remains unknown [12].

Exposure of rats to D-galactose (D-gal) has been used as a model of aging due to oxidative stress and liver damage [13]. *B. longum* and *B. animalis* reduced the expression of inflammatory cytokines and the level of malondialdehyde (MDA) and enhanced antioxidant enzymes in D-galactose-treated mice [14]. D-gal influenced the composition of the gut microbiota by increasing *Firmicutes* and reducing *Bacteroidetes* [15]. An increase in the *Firmicutes*/*Bacteroidetes* ratio was related with microbiome in oxidative stress-related disorders in Alzheimer’s patients [16], Parkinson’s disease [17], and aging [18]. Administration of *L. plantarum* Y44 reduced the *Firmicutes*/*Bacteroidetes* ratio induced by D-gal injection in rats [19].

*Bifidobacterium animalis* MSMC83 displayed antioxidant abilities and general probiotic properties in vitro. *B. animalis* MSMC83 scavenged hydroxyl free radicals and 2,2-diphenyl-1-picrylhydrazyl (DPPH) free radicals and reduced intracellular free radical levels in Caco-2 cells [20]. Therefore, this study was designed to assess the antioxidant potential of *B. animalis* MSMC83 in D-gal-induced oxidative stress in rats. To that effect, the activity of SOD, catalase, GSH-Px and MDA were quantified in the serum and liver, and TNF- α and liver enzyme (AST, ALT) levels were detected to measure liver damage. Moreover, intestinal microbial composition was analyzed in fecal samples.

## 2. Materials and Methods

### 2.1. Bacterial Strains

*Bifidobacterium animalis* MSMC83 (provided by Center Of Excellence In Probiotics, Faculty of Medicine, Srinakharinwirot University, Bangkok, Thailand) was cultivated in de Man–Rogosa–Sharpe (MRS) (HiMedia Lab., Mumbai, India) medium for 24 h at 37 °C under anaerobic conditions. The bacterial density was adjusted to 10^9^ colony forming units (CFU)/mL by measuring an absorbance of 1.3–1.5 at 600 nm. In this study, the concentration of probiotic used was the same as in the research of Xia C. et al. (2020) [14], and *B. animalis* MSMC83 at the concentration of 10^9^ CFU/mL was able to scavenge DPPH radicals and hydroxyl radicals, as well as reduced intracellular free radical levels in Caco-2 cells.

### 2.2. Experimental Design

Eight-week-old male Sprague Dawley rats (260 to 280 g) were purchased from Nomura Siam international Co., Ltd. (Bangkok, Thailand). The rats were maintained on a 12 h light/12 h dark cycle at a constant temperature (22 ± 1 °C) and were allowed access to food and water ad libitum. The committee for experimental animals of Srinakharinwirot University approved all experimental procedures (COA/AE-016-2562), and the procedures complied with the guidelines for the care and use of laboratory animals.

**Figure 1:**
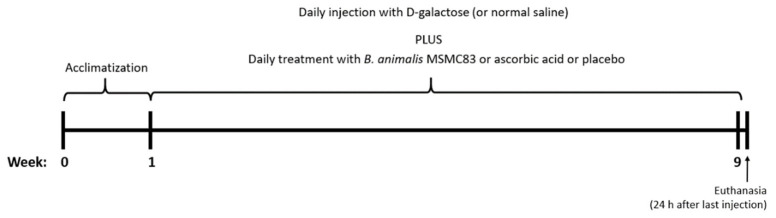
Visual presentation of experimental layout to induce accelerated oxidative stress via daily injections of D-galactose and administration of probiotic *B. animalis* MSMC83 or ascorbic acid.

After 1 week of acclimatization to the laboratory environment, rats were randomly divided into 4 groups of 5 individuals. The control group received normal saline daily (20 mL/kg body weight) via oral gavage. The D-galactose (Sigma Aldrich Co., St. Louis, MO, USA) group was treated with normal saline via oral gavage and subcutaneously injected with 5% (*w*/*v*) D-gal dissolved in normal saline (500 mg/kg body weight) once a day for 8 weeks. The D-gal plus probiotic group received the *B. animalis* MSMC83 by oral gavage at a concentration of 10^9^ CFU/day and was injected daily with 5% (*w*/*v*) D-gal, as described earlier. The last group was treated with ascorbic acid (1.0 mg/mL, Sigma Aldrich Co., St. Louis, MO, USA) and injected with 5% (*w*/*v*) D-gal at the same frequency. The experimental protocol is presented in Figure 1.

### 2.3. Sample and Organ Collections

Twenty-four hours after the final administration, rats of all groups were weighed and anesthetized using isoflurane. Subsequently, blood samples of each individual were collected via cardiac puncture, kept in heparin-ethylene diamine tetraacetic acid (EDTA)-sodium chloride (NaF) vials and stored at −80 °C for later antioxidant activity analysis. The liver, intestine, stomach, spleen, and visceral adipose tissue were all removed, weighed and frozen. The livers’ right lobes were fixed with 4% paraformaldehyde in PBS. Liver was homogenized in PBS (10% *w*/*v*) on ice using an ultrasonic homogenizer (Sonoplus HD 2070; Bandelin, Germany) at 25–30% power. The homogenates were then centrifuged at 3000× *g* for 10 min at 4 °C, and the supernatants were kept frozen to measure antioxidant and inflammatory activity.

### 2.4. Serum Biochemical Estimation

Plasma samples were collected in heparin-EDTA and in NaF tubes after anesthetizing the animals and obtained by centrifugation at 3000× *g* for 10 min at 4 °C. Each NaF blood sample was used to measure levels of glucose by the hexokinase method. Each heparinized blood sample was used to measure the levels of total cholesterol, triglycerides, high density lipoprotein-cholesterol (HDL-C), low density lipoprotein-cholesterol (LDL-C), aspartate transaminase (AST) and alanine aminotransferase (ALT). Plasma total cholesterol and triglyceride were measured by the enzymatic colorimetric method, HDL-C and LDL-C were measured by the homogeneous enzymatic colorimetric method, and AST and ALT were measured by the International Federation of Clinical Chemistry (IFCC) method without pyridoxal-5′-phosphate, according to the manufacturer’s protocols (Roche Diagnostics, Professional Laboratory, Bangkok, Thailand).

### 2.5. Histological Analysis

Fixed liver biopsies (4% paraformaldehyde in PBS, 4 °C) were dehydrated in increasing concentrations of ethanol (50%, 70%, 90%, and 100%), embedded in paraffin, and sliced into 5–7 µm sections before being mounted. Subsequently, samples were deparaffinized, cleaned repeatedly with xylene and rehydrated using a previously described method [21]. For Oil Red O staining, as described by Guo D. et al. (2022) [22], frozen liver sections were fixed with sucrose and stained with Oil Red O (Sigma Aldrich Co., St. Louis, MO, USA). Eventually, slides were stained with hematoxylin and eosin (H&E). Morphology was observed under a light microscope (OlympusBX53, Olympus Corporation, Tokyo, Japan) and photographs were made.

### 2.6. Determination of TNF-α Level in Liver

The presence of TNF- α in the supernatant of liver homogenates was measured using a rat sandwich ELISA kit (R&D Systems, Minneapolis, MN, USA). The TNF-α levels were expressed in pg/mL using a standard curve.

### 2.7. Measurement of Oxidative Stress Markers in Plasma and Liver

The enzymatic activities of SOD, catalase, GSH-Px, and MDA concentrations in the plasma and liver homogenates were measured by chemical colorimetric analysis with a SOD assay kit (Cayman Chemical, Ann Arbor, MI, USA), a catalase assay kit (Cayman Chemical, Ann Arbor, MI, USA), a GSH-Px assay kit (Cayman Chemical, Ann Arbor, MI, USA), and a thiobarbituric acid reactive substances assay kit (Cayman Chemical, Ann Arbor, MI, USA), following the manufacturer’s protocols. Each enzyme activity was quantified using a standard curve and expressed as U/mL, while the concentration of MDA was expressed as nmol/mL.

### 2.8. Analysis of Gut Microbiota by Next-Generation Sequencing

For gut microbiota analysis by next-generation sequencing, fecal samples were collected 24 h after the last injection while animals were anesthetized. Briefly, fecal DNA was extracted using a QIAamp Fast DNA Stool Mini Kit (Qiagen, Hilden, Germany). Extracted DNA concentration was measured with a Nanodrop 2000 spectrophotometer (Thermo Scientific, Waltham, MA, USA), and fragment size was evaluated by agarose gel electrophoresis. The V3 and V4 hypervariable regions of the 16S rRNA gene were amplified from extracted DNA by Illumina Miseq sequencer (Illumina, San Diego, CA, USA) [23]. The universal primers for Miseq PE300 sequencing used in this study were as followed: PE read 1 sequencing primer: 5′ ACACTCTTTCCCTACACGACGCTCTTCCGATCT 3′ and PE read 2 sequencing primer: 5′ CGGTCTCGGCATTCC TGCTGAACCGCTCTTCCGATC 3′. The MiSeq/Novaseq control software (Illumina, San Diego, CA, USA) was used to read sequence information. Sequences were clustered into operational taxonomic units (OTUs) using VSEARCH clustering (1.9.6) based on at least 97% sequence similarity. Then, the sequence of 16S rRNA was analyzed using Silva 132 database. Then, the Ribosome Database Program (RDP) classifier Bayesian algorithm of representative sequences analyzed the OTU species taxonomy. Based on OTU calculation and analysis, the results were obtained and expressed as Shannon Venn diagram and the community relative abundance.

### 2.9. Statistical Analysis

The statistical analysis was performed using Prism software version 8.02 (GraphPad Software; San Diego, CA, USA). Data are expressed as means ± SD. The statistical significance of data comparisons was determined using one-way analysis of variance (ANOVA), followed by Tukey’s multiple comparison tests. A value of *p* < 0.05 was regarded as statistically significant.

## 3. Results

### 3.1. Changes in Body and Organ Weight

The body and organ (liver, spleen, stomach, and visceral adipose tissue) weights of rats were recorded as shown in Table 1. The body weight of rats given D-gal and *B. animalis* MSMC83 or ascorbic acid was significantly decreased compared to D-gal alone (*p* = 0.0155, 0.0189). The liver weight of D-gal rats increased significantly compared to the control group (*p* = 0.0290), while treatment of D-gal animals with *B. animalis* MSMC83 or ascorbic acid decreased liver weight significantly (*p* = 0.0078) to levels comparable to that of the control group. No significant differences in spleen and stomach measurement were observed. Interestingly, the adipose tissue weight following D-Gal treatment was significantly increased compared to that of the control group, but not in the D-Gal + *B. animalis* or ascorbic acid groups (*p* = 0.0002, 0.0007). Thus, *B. animalis* MSMC83 counteracted the increases in body, liver, and adipose tissue weight during D-galactose-induced oxidative stress.

### 3.2. Biochemical Changes in Plasma

Fasting blood glucose levels, lipid profiles, and tests of liver function measured in the plasma of the different groups are shown in Table 2. A significant increase in glucose, total cholesterol triglyceride and AST levels was observed in D-gal rats compared to the control group (*p* < 0.05). Treatment with *B. animalis* MSMC83 restored the levels of glucose (105.00 ± 3.46 mg/dL), total cholesterol (50.33 ± 1.53 mg/dL), triglycerides (128.00 ± 5.57 mg/dL), and AST (68.07 ± 3.90 U/L) to levels comparable to those of the control group and significantly lower compared to those of the D-gal group (*p* < 0.05). Interestingly, treatment with the probiotics reduced the glucose levels further than treatment with ascorbic acid (*p* < 0.0001), while the content of total cholesterol and AST were similar in both groups (*p* > 0.05). Therefore, feeding with *B. animalis* MSMC83 provided an efficient means to decrease levels of glucose, total cholesterol triglyceride and AST during D-galactose-induced oxidative stress.

### 3.3. Effect of B. animalis MSMC83 on Liver Inflammation

**Figure 2:**
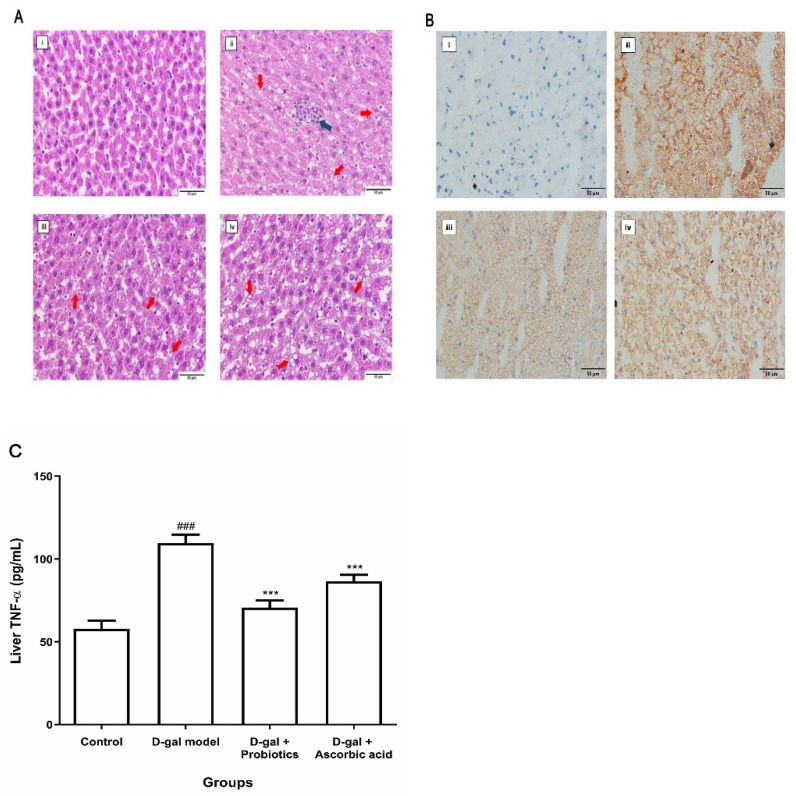
Protective effect of *B. animalis* MSMC83 and ascorbic acid on liver inflammation. Liver histopathological alterations with H&E staining (**A**) and Oil Red O staining (**B**), magnification 400× *g*. Control group (**i**), D-gal model group (**ii**), D-gal plus probiotic group (**iii**), D-gal plus ascorbic acid group (**iv**). Red arrows indicate macrovesicular fat droplets; blue arrow indicates inflammatory infiltration. (**C**) Tumor necrosis factorα in oxidative stress rats. Data are presented as means ± SD (*n* = 5). ### *p* < 0.001 as compared with the control group. *** *p* < 0.001 as compared with the D-gal model group.

The noticeable change in AST and ALT levels in D-gal rats treated with *B. animalis* suggested decreased damage and inflammation in livers of the probiotics group. Therefore, liver biopsy samples were stained with hematoxylin and eosin (H&E) for histological analysis (Figure 2A). Livers from the control group (Figure 2A, panel i) were of normal size, and no abnormalities in the morphological structure were observed. D-gal injection resulted in hepatocellular swelling, vacuole degeneration, disorganized structures, numerous lipid accumulations, and moderate inflammatory cell infiltration (Figure 2A, panel ii). Interestingly, *B. animalis* MSMC83 treatment reduced hepatic inflammation caused by D-galactose (Figure 2A, panel iii) in a fashion similar to the treatment with ascorbic acid (Figure 2A, panel iv). The Oil Red O staining in all groups confirmed lipid accumulation in liver (Figure 2B, panel i–iv). In order to further assess the level of inflammation in the liver, concentrations of TNF-α, a pro-inflammatory cytokine expressed in the liver, were measured. As expected, the TNF- α concentration increased to 109.60 ± 5.11 pg/mL in D-gal-exposed rats as compared with the control group (Figure 2C, *p* < 0.0001). The treatment of the animals with ascorbic acid led to a decrease in expression of TNF- α, and treatment with *B. animalis* MSMC83 also reduced the TNF-α concentration, to 70.67 ± 4.22 pg/mL (*p* < 0.0001). Therefore, administration of *B. animalis* MSMC83 could mitigate D-galactose-induced liver inflammation.

### 3.4. Effect of B. animalis MSMC83 on Oxidative Stress Markers in Plasma and Liver

**Figure 3:**
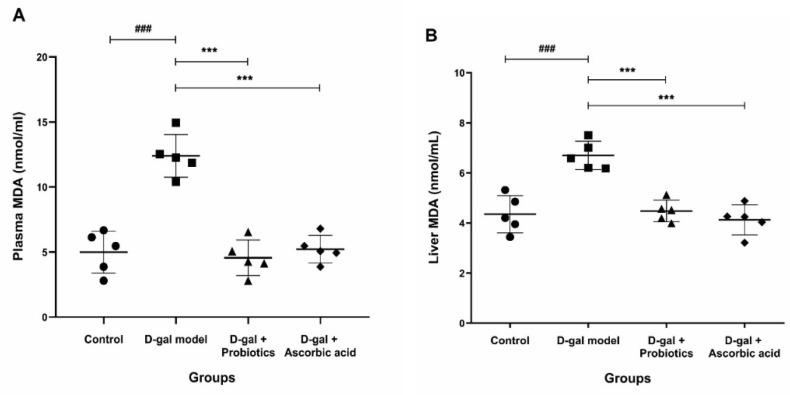
Effect of *B. animalis* MSMC83 or ascorbic acid on the plasma and liver malondialdehyde (MDA) levels in oxidative stress rats. Plasma MDA (**A**), liver MDA (**B**). ●, ■, ▲, ◆ as individually in each group. ### *p* < 0.001 as compared with the control group. *** *p* < 0.001 as compared with the D-gal model group (*n* = 5).

We next tested whether treatment with *B. animalis* MSMC83 add a systemic effect. The presence of malondialdehyde (MDA), a final product of lipid peroxidation by free radicals, is a powerful biological indicator of systemic oxidative stress [24]. As expected, the MDA level after D-gal treatment was significantly increased compared to its level in the control group (Figure 3A). *B. animalis* MSMC83 and ascorbic acid feeding decreased the level of MDA in plasma significantly, to 4.56 ± 1.37 nmol/mL and 5.23 ± 1.06 nmol/mL, respectively (*p* < 0.0001). Similarly, the hepatic MDA content in the D-Gal group was significantly higher than that in the control group (*p* < 0.0001, Figure 3B), and it was significantly reduced by the intervention with probiotics (4.48 ± 0.43 nmol/mL, *p* = 0.0001), to levels comparable with those of the control and ascorbic acid groups (*p* > 0.05). Therefore, *B. animalis* MSMC83 treatment reduced lipid peroxidation to levels comparable with the positive control group and returned it to normal levels.

**Figure 4:**
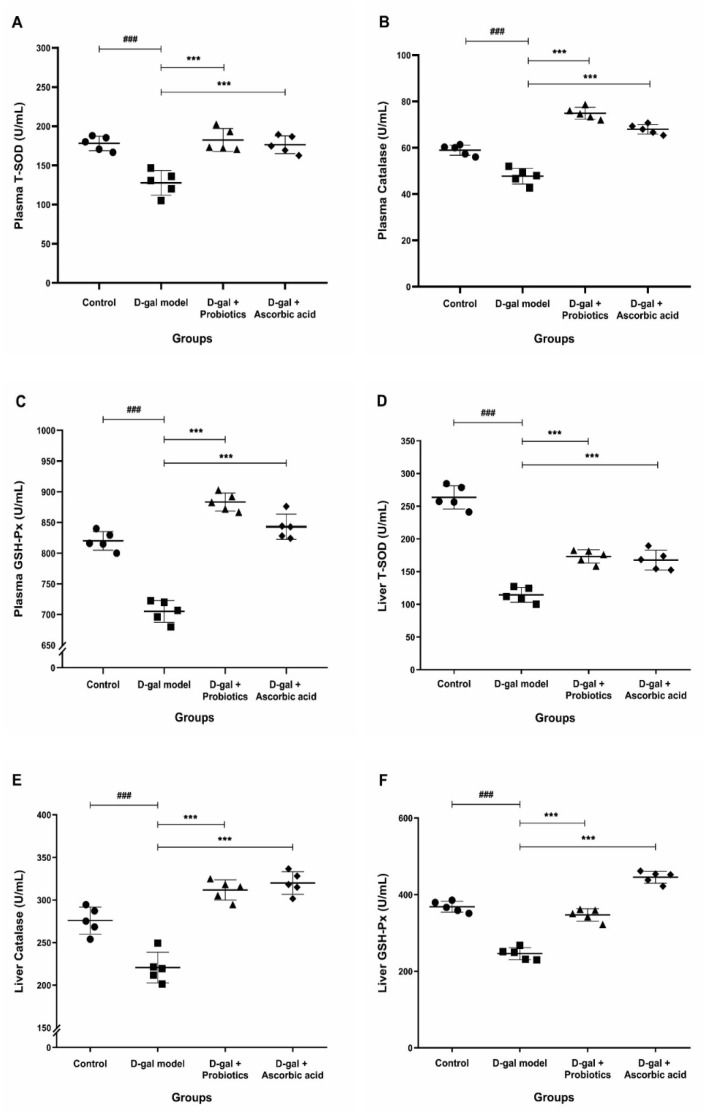
Effect of *B. animalis* MSMC83 or ascorbic acid on the plasma and liver antioxidant index in oxidative stress rats. Plasma superoxide dismutase; SOD (**A**), plasma catalase (**B**), plasma glutathione peroxidase; GSH-Px (**C**), liver SOD (**D**), liver catalase (**E**), liver GSH-Px (**F**). ●, ■, ▲, ◆ as individually in each group. ### *p* < 0.001 as compared with the control group. *** *p* < 0.001 as compared with the D-gal model group (*n* = 5).

We hypothesized that the reduced levels of inflammation (Figure 1) and MDA (Figure 2) after *B. animalis* treatment were due to the increase in antioxidant activity. Therefore, in each group, we measured the levels of SOD, catalase and GSH-Px in the plasma and liver. The activity of the three antioxidant enzymes in the plasma and liver of the D-gal-treated animals was significantly decreased compared to that in the control group (Figure 4). However, the plasma antioxidant ability was restored for animals of the D-gal + *B. animalis* MSMC83 group (Figure 4A–C). The activity of SOD, catalase, and GSH-Px after *B. animalis* treatment increased to 182.40 ± 14.65 U/mL, 883.20 ± 14.62 U/mL, and 74.93 ± 2.56 U/mL, respectively, well above the activity of the D-gal treated group (*p* < 0.0001). Interestingly, the *B. animalis* MSMC83-treated rats exhibited improved plasma SOD, GSH-Px, and catalase activities compared to the control group. The activity of the antioxidant enzymes was also favorably increased in the plasma of animals given ascorbic acid (Figure 4A–C).

We also measured the antioxidant activity of these enzymes in the liver extract after the different treatments to elucidate the possible mechanisms responsible for the protective effect of probiotics (Figure 4D–F). Compared with the control group, hepatic antioxidant enzymatic activity was significantly decreased in the D-gal model group. In contrast, *B. animalis* MSMC83 consumption completely (catalase, GSH-Px) or partially (SOD) restored antioxidant activities to normal levels (Figure 4D–F). Therefore, *B. animalis* MSMC83 demonstrated a strong ability to restore antioxidant capacity during D-gal-induced oxidative stress by increasing the antioxidant index, partially through the reduction of liver inflammation.

### 3.5. The Dysbiosis Induced by D-gal Was Prevented by B. animalis

**Figure 5:**
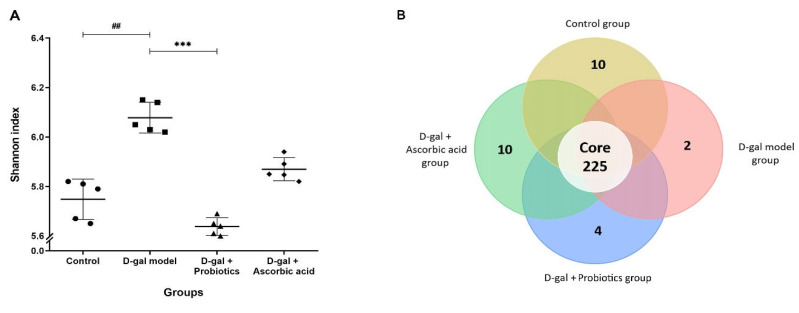

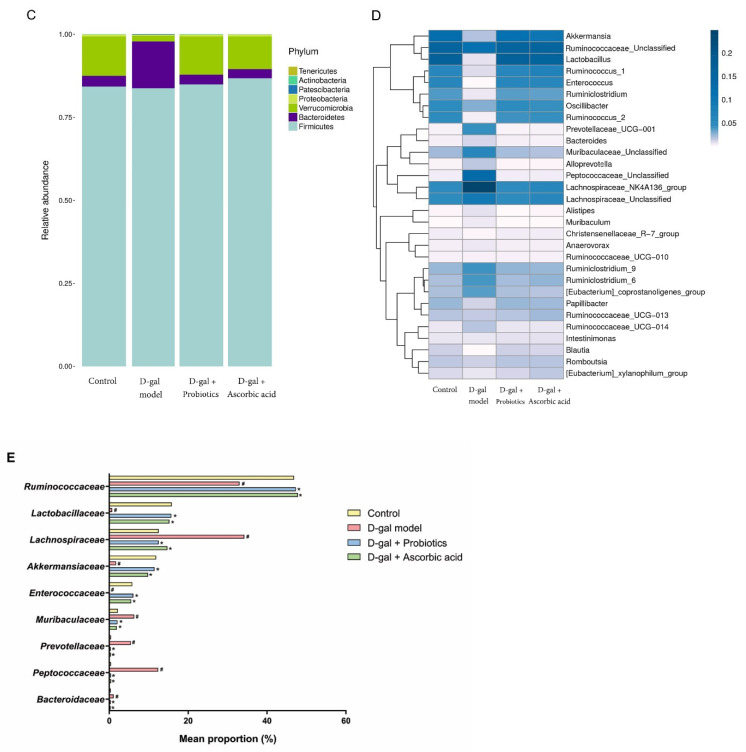
Effect of *B. animalis* MSMC83 treatment on composition of gut microbiota. Shannon index (**A**), Venn diagram representation of the number of OTUs (**B**), relative abundance of gut microbiota at the phylum level (**C**), heatmap of the gut microbiota at the genus level (**D**), and relative abundance of gut microbiota at the family level (**E**). All data are indicated as mean ± SD of 5 mice per group. ●, ■, ▲, ◆ as individually in each group. # *p* < 0.05, ## *p* < 0.01 as compared with the control group. * *p* < 0.05, *** *p* < 0.001, as compared with the D-gal model group.

To assess how treatment of D-gal rats with *B. animalis* MSMC83 affected the gut microbiota, fecal samples were analyzed. A total of 441,964 16S rDNA sequences clustered into OTUs from 20 samples (5 samples in each group) were obtained through Illumina MiSeq sequencing. The total number of sequences for each group was 110,491. The Shannon diversity index decreased significantly in the D-gal model group, indicating that the oxidative stress affected the integrity of the gut microbiota (Figure 5A). However, the use of *B. animalis* in the D-Gal group restored the diversity to a level observed in the control group (*p* = 0.0175, *p* > 0.05). As shown in the Venn diagram (Figure 5B), 225 OTUs were shared by the four groups. The D-gal model group microbiota showed two specific OTUs, and the probiotic group four OTUs, while the ascorbic acid group and the control group microbiota each contained 10 specific OTUs. The composition of the gut microbiota was examined in all groups to compare the composition of intestinal microflora at the phylum level (Figure 5C). The control, probiotic and ascorbic groups were together in a hierarchical clustering tree, while the D-gal model group was not, due to the high proportion of *Bacteroidetes* (Figure 5D). Gut microbiota phyla such as *Firmicutes*, *Bacteroidetes*, *Verrucomicrobia* and *Proteobacteria* were highly dominant in the D-gal rats. Compared with the control group, the average relative abundance of *Bacteroidetes* increased from 3.19% to 14.07% following D-gal-induced oxidative stress. Therefore, the ratio of *Firmicutes* and *Bacteroidetes* (F/B) decreased from 26.42% to 5.95%. Strikingly, feeding with probiotic *B. animalis* MSMC83 completely prevented the F/B ratio drop observed in the D-gal rats. A heatmap was generated to reflect similarities or differences in community composition at the genera level (Figure 5D). In the D-gal model group, the relative abundances of *Ruminococcaceae*, *Lactobacillaceae*, *Akkermansiaceae*, and *Enterococcaceae* were decreased compared with those in the control group. This was reversed after treatment by probiotic *B. animalis* MSMC83 (*p* < 0.05, Figure 5E). Moreover, probiotic *B. animalis* MSMC83 administration also induced enrichment in *Lachnospiraceae*, *Peptococcaceae*, *Muribaculaceae*, *Prevotellaceae*, and *Bacteroidaceae* after D-gal injection-induced dysbiosis (*p* < 0.05). 

## 4. Discussion

Oxidative stress induced by D-galactose has three different pathways. In the first pathway, high levels of D-gal lead to an increase in galactitol through a reaction catalyzed by galactose reductase. The accumulation of galactitol in cells affects osmotic pressure and causes abnormalities of the antioxidant defense system. Additionally, D-galactose can stimulate ROS production through stimulation of NADPH oxidase. D-galactose is oxidized into aldehydes and hydrogen peroxide, which can generate ROS. This pathway is an important factor in quickly accelerating the mechanisms that cause oxidative stress [25]. Previous studies have found that excessive amounts of ROS lead to oxidative stress, which has been confirmed to be a leading cause of many types of organ damage [26]. The liver produces large amounts of ROS, which lead to liver damage, and its function gradually declines [27]. Additionally, the liver is extremely responsible for detoxification, and most of the D-gal is metabolized in the liver [28]. Results for organ weight showed different liver weights among the D-gal model, probiotic, and control groups (*p* < 0.05). Body, liver and adipose tissue weights of probiotic and ascorbic acid groups decreased, compared with those of the D-gal model group (*p* < 0.05). The changes in the liver weight in the D-gal model group clearly indicated liver damage. Excessive ROS from intake of high doses of D-gal increase lipid oxidation and fat transportation into cells, causing fat deposition. As an increase in fat accumulation occurs, the body weight and total fat increase as well [29]. Li B et al. [30] showed that oral administration of *L. helveticus* KLDS1.870 alleviated the changes caused by D-gal, including reduced liver weight and liver damage. Therefore, administration of *B. animalis* MSMC83 had potential abilities in repairing the D-gal-induced liver oxidative stress damage. D-galactose contributes to the generation of ROS via reaction with amino acids to form glycation end-products through non-enzymatic glycation with the benefits of low toxicity, slow oxidation process, and no lethal effect. Moreover, oxidative stress from long-term exposure to D-gal can cause inflammation and affect insulin resistance [31]. As the level of insulin resistance increases, the amount of glucose in the bloodstream increases, resulting in more free radical formation in mitochondrial dysfunction [32]. Moreover, a biochemical marker of liver oxidative stress is the permeability of the cell membranes that affect the release of AST and ALT in the blood [33]. Our study found that *B. animalis* MSMC83 reduced fasting blood glucose and the level of plasma AST in D-gal-induced oxidative stress, while the ALT level in the probiotic group tended to decrease. The results of Yu X. et al. [34] demonstrated that ALT and AST decreased significantly in *L. mucosae* LMU1001 groups, indicating their protective effect on liver. Moreover, previous studies showed that hypertriglyceridemia and hypercholesterolemia are closely related to oxidative stress [35]. D-gal-induced oxidative stress was also associated with significant lipid metabolism changes. The levels of triglyceride and total cholesterol in the D-gal model group increased but decreased with administration of *B. animalis* MSMC83. Therefore, lowering triglyceride and total cholesterol levels by receiving *B. animalis* MSMC83 may help reduce risk factors for atherosclerosis and cardiovascular complications. Moreover, *B. animalis* MSMC83 may decrease insulin resistance and protect the structure of the hepatocellular membrane in oxidative stress [36].

The liver is the important organ of D-gal metabolism. Oxidative stress causes liver inflammation, leading to secretion of pro-inflammatory cytokines such as TNF-α, interleukin-1 beta (IL-1β) and interleukin-6 (IL-6) [37]. Moreover, polymorphonuclear neutrophils (PMNs) at the inflammation site stimulate increased ROS production. ROS also regulates the expression of genes that induce inflammation. The effect of this activation causes the release of pro-inflammatory cytokines and other substances that damage tissues and organs [38]. Therefore, we first assessed the pro-inflammatory response to liver cytokines. The levels of TNF-α was significantly increased in the D-galactose-injected control group, while the levels of TNF-α in rats receiving *B. animalis* MSMC83 were significantly decreased. Previous studies have shown that *B. longum* and *B. animalis* alleviated pro-inflammatory cytokines such as TNF-α, IL-1β and IL-6 in the hippocampus of D-gal model mice through inhibition of NF-κB and TLR4 signaling [18]. Moreover, histological examination is necessary to determine hepatic toxicity caused by the stimulation of the metabolite. When the overgeneration of free radicals is induced or the function of the antioxidant system is impaired, cells and tissues are injured by oxidative stress [39]. Probiotic *B. animalis* served a preventive role against liver damage and improved anti-inflammatory effects in oxidative stress. Treatment with *B. animalis* significantly attenuated biochemical and histopathological changes, suggesting the probiotic *B. animalis* could mitigate D-gal-induced hepatic damage.

Free radicals are eliminated through the body’s redox system via generated antioxidants, which reduces the risk factors of oxidative stress [40]. The mechanism of antioxidant modulation is to produce antioxidant metabolites, increase levels of antioxidant metabolites of the host and regulate the intestinal microbiota [8]. The SOD, catalase, GSH-Px and MDA activities were used to assess the protective effect of *B. animalis* MSMC83 in oxidative stress conditions. The results of this study showed that the activities of antioxidant enzymes in plasma increased, and the oxidation product MDA activity decreased in plasma by treatment with *B. animalis* MSMC83 in D-gal-induced oxidative stress. Remarkably, *B. animalis* MSMC83 had the same antioxidant capacity as ascorbic acid in improving plasma levels of SOD, catalase, GSH-Px, and MDA in vivo. SOD, catalase, and GSH-Px are primary antioxidant enzymes [41]. SOD is an important enzyme within cells to prevent oxidative damage and a sensitive marker of liver damage through the catalytic process of superoxide ions to H_2_O_2_ [42], whereas catalase enzymes prevent the production of highly toxic hydroxyl radicals by accelerating the decomposition of hydrogen peroxide [43]. Additionally, GSH-Px is the key antioxidant enzyme in the glutathione (GSH) system that scavenges free radicals by accelerating the reduction of hydrogen peroxide to a non-toxic product and removing highly reactive lipid peroxide in cells [44]. As SOD, GSH-Px, and catalase levels increase, ROS levels can be reduced to prevent the oxidation of proteins, fats and DNA [45]. Several probiotics can produce and secrete SOD [46]. According to the study by [47], two *L. fermentum* strains expressed generation of SOD in oxidative stress conditions. Moreover, probiotics were able to enhance the antioxidant metabolite system of the host [48]. Similarly to our findings, previous studies reported that oral administration of *L. plantarum* CCFM10 could raise SOD and GSH-Px activities and lower the level of MDA in serum [49]. The study by Xu R. et al. in 2011 [50] reported that *B. animalis* RH significantly increased SOD, catalase, and total antioxidant capacity in D-galactose-induced mouse models. Similarly to the previous study, *B. longum* BAMA-B05BAu-B1024 and *B. animalis* LPL-RH enhanced SOD and GSH-PX activities and reduced MDA activity in D-gal model mice [14]. After receiving a mixed probiotic formulation of *B. animalis* subsp. *infantis* BLI-02, *B. breve* Bv889, *B. bifidum* VDD088, *B. animalis* subsp. *lactis* CP-9 and *L. plantarum* PL-02, middle-aged rats exhibited antioxidant activity by enhancing beneficial intestinal microflora [51]. The transcription factor nuclear factor kappa B (NF-κB) responded directly to oxidative stress because ROS mediate the activation of redox-sensitive transcription factor NF-κB [52]. The extracellular polysaccharide LAB was able to inhibit NF-κB and ROS production to prevent lipopolysaccharide-induced inflammation in RAW 264.7 macrophages [53]. Similarly, *L. plantarum* FC255-treated mice expressed and translocated liver nuclear factor E2-related factor 2 (Nrf2), promoting the transcription of genes for antioxidant enzymes via the Nrf2-Keap1-ARE pathway [54]. Moreover, LAB was able to downregulate ROS-producing enzymes in male rats [55]. Additionally, consumption of probiotic *B. animalis* MSMC83 may initiate the production of intestinal ROS-inhibiting enzymes, leading to elevation of antioxidant enzymes that regulate antioxidant signaling pathways. Therefore, these results suggested that *B. animalis* MSMC83 increases intracellular antioxidant enzyme activities and reduces peroxidation product levels in oxidative stress.

An imbalance in the gut microbiota causes many health problems, such as increased oxidative stress [56], inflammation [57], and immune disorders [58]. The function of probiotics to modulate intestinal microbiota and intestinal disease has been studied [59]. Due to their specific adaptations, probiotics can compete with other microorganisms, leading to alteration and modulation of the gut microbiota [60]. Moreover, probiotics can replace, adhere to and colonize with intestinal epithelial cells [61]. Previous studies supported that oxidative stress induces the dysbiosis of gut microbiota [62]. Regulation of the gut microbiota is also one mechanism of reducing ROS [63]. Abnormal gut microbiota and excessive pathogens induce intestinal epithelial ROS generation [64]. This study found that the F/B ratio decreased after D-gal injection, which is in agreement with the study by [19]. A reduced F/B ratio was found in the intestinal microbiota of Alzheimer’s and Parkinson’s disease patients, most likely due to oxidative stress [65]. The administration of the *B. animalis* MSMC83 group reversed the changes in gut microbiota constituents by increasing *Bacteroidetes*’ relative abundance, similarly to that of the control group. Interestingly, *Lactobacillus*’ relative abundance was reversed in probiotic group, compared with its levels in the D-gal model group. *Lactobacillus* belongs to the *Lactobacillaceae*, which is capable of inhibiting pathogenic bacteria, producing acid by reducing intestinal pH, producing short-chain fatty acids (SCFAs) and maintaining intestinal microbiota balance [66]. An increase in *Lactobacillus* in the probiotic group may be indicated to promote the survival of good microorganisms in the intestine. Moreover, *B. animalis* MSMC83 administration also increased the amount of *Ruminococcaceae* and *Akkermansiaceae*. *Ruminococcaceae,* belonging to the phyla *Firmicutes*, were able to prevent the colonization of pathogens and stimulate anti-inflammatory activity of regulatory T cells by producing SCFAs [67]. *Akkermansiaceae* (phylum *Verrucomicrobia*) binds to regions within the mucous layer, which is a key mechanism in regulating the mucus turnover of the host and is, therefore, recommended as a biomarker of intestinal health. It also restores intestinal barrier function by stimulating mucus production, helps normalize the metabolism of adipose tissue and improves metabolic inflammation. Previous studies have shown that antioxidants alleviate oxidative stress by balancing the intestinal microflora. LAB has the potential to regulate homeostasis of the gut microbiota via redox signaling [68]. These results are in agreement with the study by Zhao J et al. [15]. *L. plantarum* CCFM10 and *L. plantarum* RS15-3 changed the composition of the microbiota at the phylum and genus levels after being induced by D-gal, to similarly to the control group. These findings suggested that administration of *B. animalis* MSMC83 protects against changes in gut microbiota, possibly one of the mechanisms for resistance to oxidative stress.

## 5. Conclusions

Administration of *Bifidobacterium animalis* MSMC83 in oxidative stress induced by D-galactose was able to reduce the body weight and liver and total adipose tissue weights. Glucose, cholesterol, triglyceride, and AST in plasma were also reduced. *B. animalis* MSMC83 can reduce the accumulation of fat in the liver, liver inflammation, and hepatic TNF-α levels. Additionally, *B. animalis* MSMC83 was able to increase antioxidant enzymes that consist of superoxide dismutase, catalase, and glutathione peroxidase, and decrease malondialdehyde in plasma and liver. Moreover, administration of *B. animalis* MSMC83 modulated homeostasis of gut microbiota.

## Figures and Tables

**Table 1:** Effect of *B. animalis* MSMC83 on the body and organ weights.

Index	Control	D-gal Model	D-gal + Probiotics	D-gal + Ascorbic Acid
Body weight	600.60 ± 8.79	635.67 ± 10.97	573.65 ± 16.44	576.69 ± 31.60
Liver	14.90 ± 0.35	17.05 ± 1.37 ^#^	14.03 ± 0.34 **	15.08 ± 0.49 *
Spleen	0.82 ± 0.05	0.93 ± 0.03	0.77 ± 0.05	0.83 ± 0.13
Stomach	2.75 ± 0.17	2.91 ± 0.07	3.13 ± 0.27	2.72 ± 0.60
Adipose tissue	38.11 ± 0.64	42.81 ± 0.57 ^##^	34.03 ± 1.23 ***	34.56 ± 1.73 ***

Each value is expressed as mean ± SD (*n* = 5). ^#^ *p* < 0.05, ^##^ *p* < 0.01 as compared to the control group. * *p* < 0.05, ** *p* < 0.01, *** *p* < 0.001 as compared with the D-gal model group.

**Table 2:** Effect of *B. animalis* MSMC83 on biochemicals in plasma.

Biochemical	Control	D-gal Model	D-gal+ Probiotics	D-gal + Ascorbic Acid
Glucose (mg/dL)	203.67 ± 1.53	221.33 ± 5.13 ^##^	105.00 ± 3.46 ***	172.67 ± 6.43 ***
Total cholesterol (mg/dL)	49.33 ± 3.22	60.67 ± 0.58 ^##^	50.33 ± 1.53 ***	50.67 ± 2.08 ***
Triglyceride (mg/dL)	84.33 ± 3.79	229.67 ± 7.57 ^##^	128.00 ± 5.57 ***	102.00 ± 5.20 ***
HDL-C (mg/dL)	31.33 ± 5.03	32.33 ± 1.53	35.67 ± 0.58	30.33 ± 1.53
Direct LDL-C (mg/dL)	5.00 ± 1.00	5.33 ± 0.58	5.67 ± 0.58	6.67 ± 0.58
AST (U/L)	74.00 ± 4.58	90.67 ± 5.77 ^##^	68.07 ± 3.90 **	70.37 ± 9.02 *
ALT (U/L)	26.67 ± 0.58	28.33 ± 4.04	25.67 ± 3.06	27.77 ± 1.55

Each value is expressed as mean ± SD (*n* = 5). ^##^ *p* < 0.01 as compared to the control group. * *p* < 0.05, ** *p* < 0.01, *** *p* < 0.001 as compared with the D-gal model group.

## Data Availability

The data are contained within this article.
